# Simulation training in radiation oncology

**DOI:** 10.1002/acm2.70345

**Published:** 2025-12-18

**Authors:** Michael C. Schell, Andrew W. Beavis, Derek W. Brown, Michael Milano, Michael O'Neill, Yan Yu, Daniel C. Pavord, Peter Dunscombe

**Affiliations:** ^1^ University of Rochester Medical Center Rochester New York USA; ^2^ Queens Centre for Oncology & Haematology Cottingham UK; ^3^ University of California, San Diego La Jolla California USA; ^4^ University of Rochester Rochester New York USA; ^5^ Alan B Pearson Regional Cancer Center Lynchburg Virginia USA; ^6^ Radiation Oncology Thomas Jefferson University Kimmel Cancer Center Philadelphia USA; ^7^ Allegheny General Hospital Pittsburgh Pennsylvania USA

**Keywords:** competency, safety, simulation training

## Abstract

Simulation training can be used in the design of new processes or, more commonly, to train and re‐train healthcare personnel in standard processes. Simulation training is one avenue to making radiation oncology a high reliability health care intervention. In this paper, we explore the relevance of simulation training to the provision of high‐quality and safe radiation oncology practice against the background of simulation training conducted in high reliability industries. The use of simulation training in healthcare, as well as specific examples in radiation oncology, is reviewed. An outline of the steps for developing a simulation training program is provided. Simulation training is critical for the verification of training and treatment delivery. As such, it increases the integrity and transparency of patient care.

## 1 SIMULATION TRAINING: AN OPPORTUNITY FOR RADIATION ONCOLOGY

The expression “simulation training”, in our context, encompasses approaches to the development and maintenance of competence, in both routine and unexpected situations in a clinical process, and which take place in the absence of an actual patient. Thus, this form of training simulates the urgent demands of clinical situations but without the risk of patient harm. Simulation training can be used in the design of new processes or, more commonly, to train and re‐train health care personnel in standard processes. Simulation training is one avenue to making radiation oncology a high reliability health care intervention.^1^ In this paper, we explore the relevance of simulation training to the provision of high‐quality and safe radiation oncology practice against the background of simulation training conducted in high reliability industries.

The collective failings of the medical community in the area of patient safety were brought dramatically to light in the pivotal document “To Err is Human: Building a Safer Health Care System”.^2^ This document reported the astonishing estimate of 44 000 to 98 000 deaths per year in the United States (U.S.) from avoidable adverse medical events. While the majority of these unnecessary deaths were likely due to medication errors and nosocomial infections, potentially fatal errors in radiotherapy planning and delivery are also known to occur. A 2023 Canadian study found that 1 in 17 hospital stays resulted in at least one harmful event.^3^ In spite of the fact that we, in radiation oncology, exert great effort to ensure the safety of our patients, well‐publicized events, particularly of the last two decades, have shown that we are not always successful. A valuable summary and an analysis of deaths and near‐miss events, due to errors in radiotherapy procedures, have been presented in the comprehensive report by the World Health Organization “Radiotherapy Risk Profile”.^4^ Consequently, simulation training is a tool that we should use to make radiotherapy safer and of higher quality.

Traditionally, in the radiotherapy professions, education and training leading to the development of the necessary competencies have been delivered in two generally complementary formats. The first level is primarily didactic (i.e., in the classroom and in the absence of the patient). The second, practical level, intended to consolidate and build on classroom education, takes place “in the field,” that is, treating patients or commissioning equipment for immediate clinical use, with mentorship from licensed, credentialed practitioners. While such approaches have served the community and patients well, they are no longer adequate in an increasingly complex and rapidly changing environment. Practical training is typically completed once and is rarely repeated to safeguard against bad habits slowly supplanting the “best practice” clinical process. As we will discuss, high reliability industries have recognized and addressed this shortcoming of practical training.

One‐time training “in the field” suffers from these serious limitations:

1. The timelines for completing tasks in the clinical environment can compromise the learning experience, as the priority is frequently to complete the task rather than impart knowledge to and develop competence in the trainee.
2. Different teachers adopt different approaches to a particular clinical task, a practice that does not facilitate the standardization that is recognized within and outside high reliability industries as one of the important keys to an intrinsically safe operation.^1^
3. Finally, and critically, in most actual situations in the clinic, it is not possible to demonstrate failure modes and hence develop the skills to recognize and address unusual situations. In this context, we note that vendor training focuses on performing the process correctly. Vendors rarely train the user to recognize failures and errors.

Though hospitals are classed as high reliability organizations, medical error constitutes one of the leading causes of avoidable death in the United States.^5^ One of the characteristics of a high reliability organization is its development and cultivation of a positive risk culture^6^, ^7^ An analysis was performed to compare the risk cultures in radiation oncology, nuclear power, and aviation to discern factors that reduce avoidable errors in high reliability organizations. Consultation with subject matter experts aided in the development of a corpus of seminal risk management papers in each field. Using the principles of network theory, a bibliographic coupling analysis studied the connections between the fields and the extent to which each field is learning from another's best practices in risk management and culture. The major conclusion is that nuclear power and aviation risk cultures are tightly coupled, and the risk culture in radiation oncology lags behind the previous two cultures.^8^ This could be due to several factors: (1) radiation oncology has no independent national regulatory authority, which the other two fields have; (2) radiation oncology has no mandatory training protocols, including no mandatory simulation training (again, unlike nuclear power and aviation), and (3) radiation oncology has no mandatory staffing levels. Nuclear power has zero avoidable deaths per year, aviation has up to 500 deaths annually, and medicine is estimated to have 240 000 avoidable deaths per year.^9^, ^10^, ^11^, ^12^ Data from the Radiation Oncology Incident Learning System (ROILS) show that 3% of incidents lead to an incorrectly delivered treatment, ^13^ Even the estimates of avoidable deaths per year from medicine vary widely, which itself emphasizes the need for greater transparency, training, and new regulations.

The expression “simulation training” can be interpreted to include any means of individual and team competency development that does not suffer from the three limitations described above. Given an appropriate simulation training format, the training could be repeated, reproducibly, at any time without disrupting the clinic.

It is worth reflecting that where training is performed by tutors or mentors, simulation training also removes a conflict between their teaching requirements and associated clinical responsibilities. In a review, Lane et al suggest this reduces strain on the trainers as well as the trainee, thus contributing to a safer training environment in a multi‐dimensional manner.^14^

## 2 SIMULATION TRAINING IN HIGH RELIABILITY ORGANIZATIONS

Many fields have utilized simulation training in order to achieve and maintain competency of trainees and staff during routine as well as unanticipated situations. We focus on two acknowledged high reliability industries—the commercial airline industry and the nuclear power industry. While simulation training is not the only major safety initiative in either of these industries, it constitutes a key component of their overall safety programs.

Simulation training for airline flight crews has been in place for over 88 years since the Link trainer was manufactured by Edwin Link in 1927. Of particular note is that training for pilots is ongoing, in stark contrast to our one‐off approach. Commercial pilots have biannual recurrent training sessions, spending at least 5 h per session in a simulator. A pilot, therefore, spends a minimum of 10–12 h per year.^15^ Interestingly, pilots train in pairs and use the crew concept.^16^

As has frequently been pointed out, the use of the airline industry as a comparator for the clinical activities in which we engage, particularly in regard to the delay between an error and its consequences, is of limited validity. Nonetheless, the rapid identification and correction of actual and potential system failures or hazardous conditions is a common area of concern if passengers are to arrive safely or, in our case, patients are to come through their treatment unharmed and with optimum outcomes.

It should be noted that one of the most recent airline disasters, the Boeing 737 Max failures, was partially enabled by the lack of proper simulation training. In fact, the component that caused the failure was not fully implemented in the simulators, and mention of this updated system was removed from the training manuals.^17^, ^18^

Simulation training is also used extensively in the commercial nuclear power industry, with roots in the US Nuclear Navy in 1953. In 1979, the Kemeny Commission^19^ reviewed the Three Mile Island Nuclear Power Plant accident.

Responding to this review, the Commercial Nuclear Power Industry began implementing full‐scale, immersive simulation training and radically improving nuclear power plant operations, and thus became a Highly Reliable Organization.″

Against this background of the commitment of high reliability industries to ongoing training, and in particular simulation training, it is of interest to inquire as to the status of comparable activities within the spheres of medicine in general and of radiation oncology in particular.

Simulation training has been used in NASA. The well‐known solution used to return the Astronauts safely, during the Apollo 13 mission had been worked out following a training simulation for Apollo 10, in which the Lunar Module was needed for survival, but could not be powered up in time. In essence, the practiced simulation exercise (akin to radiation contingency planning) to pre‐empt dealing with system failure proved invaluable in addressing a scenario anticipated but considered unlikely.^20^, ^21^

## 3 SIMULATIONS IN HEALTHCARE

For many years, the only simulation in health care that was widely adopted was the emergency evacuation of a medical facility necessitated by fire or some other potentially disastrous event. Emergency life‐saving courses were relatively early adopters of simulation for certification and renewal, and now many disciplines have embraced its model.

However, as shortcomings in patient safety and quality of care are identified, simulation has become a valued tool to allow trainees “to diagnose and manage both common and rare diseases, practice high‐risk procedures, and improve skills such as teamwork and communication in a safe learning environment.”^22^

In recognition of the value of simulation training, the Accreditation Council for Graduate Medical Education (ACGME) has encouraged such an approach as an instrument to demonstrate and teach its six domains of clinical medical competence—knowledge, patient care, professionalism, communication and interpersonal skills, practice‐based learning and improvement, and systems‐based practice.^23^

The modern use of simulation in health care was pioneered in the field of Anesthesiology.^23^ The improvement in the safety of anesthesia has been remarkable, with part of the improvement attributable to simulation training. The morbidity rate has decreased by a factor of 10 since the 1980s.^24^

Medical simulators and simulations are now quite diverse in their functions and capabilities, ranging from complete immersive exercises running Advanced Cardiovascular Life Support (ACLS) codes to task‐oriented practices such as improving shoulder arthroscopy. However, the goals and purposes of these simulations remain to be the enhancement and maintenance of safe, high‐quality medicine. Very thorough and detailed histories have been written about the evolution and development of different types of medical simulators and the field of medical simulation in general.^25^, ^26^ Simulation societies have emerged, and an academic journal *Simulation in Healthcare* was established in 2006.

Simulation training in healthcare is not limited to technical skills. It has also been used to improve non‐technical skills such as teamwork, communication, and situation awareness.^27^ It can also be used for quality improvement.^28^

Although the value of simulation training in medicine is increasingly recognized, it is, however, not widespread. Based on the experience of the authors, ongoing training, for those facilities that offer it, is typically 2 days per year for a few departmental clinicians rather than for entire departments. The commitment of the health care industry to simulation training is considerably less than that of the recognized high reliability industries discussed above.

## 4 SIMULATIONS IN RADIATION ONCOLOGY

The only routine simulation training exercises that occur in a typical radiation oncology department are performed for “contingency planning” and often to satisfy legal requirements (IRR2017 UK legislation). For example, emergency opening of a stuck treatment vault shielding door and emergency retraction/removal of an HDR source, which can be simulated with the dummy source during source replacement. Both these situations can present immediate hazards for the patient. However, there are other scenarios in which a failure can lead to catastrophe within seconds or less, for example, short, high‐dose treatments such as SRS. Furthermore, simple uncaught errors may lead to systematic errors where a series of patients may be mistreated over a period of time. Examples include selection of inappropriate detectors or process errors in the application of a calibration protocol such as TG51, TRS398, TRS483, or IPEM2020. Just as in the high reliability airline and nuclear power industries, such processes could be made safer with the implementation of well‐thought‐out simulation training. These simulation‐based training sessions would be performed outside of the busy clinical environment and available at any time of the day (rather than outside clinical hours), thereby facilitating quality training time and environments.

Another set of safety‐critical procedures, albeit generally with less immediate effect on patients, is equipment commissioning. A failure at commissioning is a systemic failure that affects a large cohort of patients. As commissioning happens infrequently and equipment design may have undergone major changes, previous experience, even if remembered, may no longer be completely relevant. The opportunity to access simulation training, emphasizing possible failure modes, prior to embarking on time‐constrained equipment commissioning would undoubtedly be of value in minimizing the probability of an error being made.

In high reliability industries, the focus of simulation training is on avoiding catastrophic accidents. In radiotherapy, we need, additionally, to be particularly cognizant of the fact that sub‐optimal treatment, although not necessarily leading to a catastrophe in the sense of immediate death, may well compromise the outcome for the patient. Unacceptable side effects or reduced lifespan may be the consequences of sub‐optimal cancer therapy. Any measure, including simulation training, which enhances quality and increases safety at any time in the future, is worthy of consideration for implementation. While individual elements of simulation training have been implemented, the development of comprehensive simulation training programs is very limited.

## 5 TOOLS FOR SIMULATION TRAINING IN RADIATION ONCOLOGY

The simplest and most accessible means, which could be used to identify and address certain classes of failure in a somewhat standardized process, are paper‐based exercises. For example, a suite of treatment plans could be generated, some of which contain deliberate errors. An example of the implementation of such a test suite is described below. The trainee and experienced practitioner would be expected to achieve pre‐determined levels of sensitivity and specificity in identifying errors. Similar exercises could be envisaged for Linac and treatment planning system commissioning. Devising realistic scenarios containing subtle errors requires some effort, more than might be available in many treatment centers. This writing group has already started to explore this opportunity. A centralized repository of such exercises is an efficient approach to the facilitation of the widespread adoption of this simplest form of simulation training.

Harnessing modern communications technology presents further opportunities. i.treatsafely (i.treatsafely.org) is a radiation oncology‐specific video hosting service with multiple objectives. Primary amongst these is training in clinical processes at the point of need and under the control of the trainee. Adoption of posted processes can be expected to promote standardization of practice, which has been identified as a strong intervention for patient safety (Hierarchy of Actions).^1^ Videos can be associated with quizzes, thus lending themselves to the sort of simulation training we are discussing here. This is also a simulation training avenue that is being explored.

Virtual reality presents an interactive, immersive opportunity wherein basic concepts can be illustrated and end‐to‐end processes can be simulated, akin to the “flight simulator” used in aircraft pilot training. The Virtual Environment for Radiotherapy Training (VERT) system is an example of such a simulation system available for Radiation Therapy training.^29^ It is an immersive life‐size virtual environment radiotherapy treatment room that provides trainees with fully articulated and interactive (OEM faithful linac and proton gantry models, enhanced visualization, and training aids for treatment of virtual patients in a virtual treatment room.^30^ The system has been found to increase novice radiation therapists knowledge, understanding, skills, and confidence in using linear accelerators as well as to increase enjoyment in the learning process.^31^ Whereas, originally designed to address shortfalls in Therapist training in the United Kingdom, it currently addressees multi‐professional use, including Medical Physicist training.^32^, ^33^, ^34^ It has also been reported as being used as a useful tool for ‘patient education’, to prepare them for their treatment experience.

The use of such a system is multi‐faceted; it is used to illustrate basic concepts (e.g., isocenter, set up procedures, beam delivery, dosimetry), clinical techniques (2D, 3D‐CRT, IMRT), workflow (IGRT, Quality Control (QC) processes), including end‐to‐end detailed technical simulations (ion chamber, Linac calibration protocols). To these ends, DICOM data from treatment planning systems are uploaded to create patient models and to simulate any treatment technique of the user's choice. Beam models are provided such that Physics QC processes (e.g., beam output, scanning water phantom function, small field dosimetry, and ion chamber/ linac output calibration protocols) can be undertaken in the virtual environment. In summary, the range of features available in such a system as VERT provides an environment where clinical procedures can be usefully and faithfully simulated to practice clinical skills away from the clinic. However, a key aspect to enhancing the trainees' understanding of concepts and the requirements for safe and effective clinical practice is experiential learning from having made errors or seen erroneous operation of the equipment. Computer simulation‐based systems are ideally suited to provide this experience. Within VERT, it is possible to introduce patient setup errors, which enable the user to explore the requirements of treatment accuracy and the implications of an incorrect or poor setup. Imaging (kV, Cone Beam CT) can be used to detect and correct the mispositioning, and the effects on delivered dose can be explored (dose distribution, Dose Volume Histogram comparisons). The Linac can be mis‐calibrated in a number of ways (e.g., laser calibration, field size, couch travel) to explore the impact of geometrical calibration errors or dosimetric errors (systematic output errors) in conjunction with user‐definable random dosimetric fluctuations to simulate measurement and output uncertainties.^35^ These errors can be injected into clinical scenarios without the trainees' knowledge. The system can be launched with a specific linac, patient (or QC) plan with preset error conditions, thereby leaving the trainee to assess the situation (as a learning exercise or competency assessment). The participants’ response to the situation can be monitored via observation or by them capturing/ recording the state of the Virtual Reality (VR) scene. Replicating the kind of (physical) simulators used in the airline industry is cost‐prohibitive and therefore unrealistic in our field. Virtual Reality may be the closest approach we can make; however, it can provide an extremely powerful training environment.

While not truly a simulation process in which practitioners are presented with different, potentially error‐prone scenarios, credentialing for cooperative group studies by irradiating benchmark phantoms can be considered an initial step in participation in simulation training. This can be done by involving the entire team in the process, with each member performing the tasks as they would for a real patient. For example, credentialing for some NRG clinical trials requires each facility to test the protocol by delivering the treatment to a benchmark phantom and submitting the data to the Imaging and Radiation Oncology Core, Houston (IROC). The facility must successfully scan the phantom, plan the treatment per protocol, and deliver the radiation treatment. The benchmark phantom data is quantitatively analyzed by IROC (http://rpc.mdanderson.org/RPC/home.htm). Facilities that do not participate in clinical trials can order the phantoms from the MD Anderson Dosimetry Laboratory (http://adcl.mdanderson.org/ADCL_MDACL/home_link.htm). This is an example of what is commonly called end‐to‐end testing (E2E). This type of simulation training is discussed in the AAPM report, Guidelines for Competency Evaluation for Clinical Medical Physicists in Radiation Oncology^36^

The American Society for Radiation Oncology (ASTRO) and the American Brachytherapy Society (ABS) teamed up for a prostate low dose rate (LDR) brachytherapy simulation workshop at ASTRO's 57^th^ annual meeting. This allowed participants actual “hands‐on” simulation with instruction and practice in pre‐implant ultrasound and post‐implant CT and MRI contouring using VariSeed (Varian) and MIM planning systems. Phantoms were implanted with “dummy seeds.” A similar process could be used by institutional teams to work through the LDR implant process and simulate potential problem scenarios.

Whichever approaches are adopted will require the allocation of resources for an effective simulation training program. One publication has documented the need for substantial resources,^37^ reinforcing the need for shared resources as described above. The questions remain: which clinical processes and which system failure modes should receive the highest priority for such training? The incident‐learning systems currently in use can help us to identify the most error‐prone activities in the clinic. This retrospective information can be augmented with prospective analysis of system safety of the type undertaken by the AAPM's TG 100.^12^ The TG 100 document describes in detail a method for identifying and hence prioritizing the most serious failures in the radiotherapy process. With incident learning and prospective quality management approaches such as TG 100, resources for simulation training can be directed towards those activities which we know are, or postulate to be, the most prone to error that compromise patient outcome.

## 6 DEVELOPING A SIMULATION TRAINING PROGRAM

There are two considerations when implementing simulation training in your clinic. The first is the scope of the simulation training program. The Society for Simulation in Healthcare (SSH) has developed a Simulation Program Policy and Procedure Manual Model Template.^38^ This document describes in detail the structure of a comprehensive simulation training program by listing what policies and procedures should be in place. The scope may be beyond what can be implemented in your department, but the concepts are instructive. Some key elements are ethics, course content, evaluation methods, feedback and debriefing, privacy, and safety.

The next consideration is designing the simulation scenarios. The scenarios could be isolated to one group within the department or could be multidisciplinary.

The outline below can be used as a guide for developing the scope of your simulation training program.

Outline for Simulation Training to maximize the integrity of patient care:

Who do you want to participate in the program?

1. Training new practitioners.
2. Routine training for proficiency and on updated equipment/software. Expect to spend 2 to 4 weeks per year on training.

How extensive do you want the program to be?

1. Training for error recognition. Design the Simulation Training procedure to test practitioners for correct clinical procedures and to detect unexpected/hidden errors.
2. Use simulation training to test and improve the clinical procedure design. Use phantoms to test the entire procedure and the entire clinical team, and evaluate the performance.
3. Use virtual training environments to increase the realism of the training.

Once these are determined, the specifics can be outlined. Topics to be addressed as described in the SSH document include:

1. General information such as mission and vision statements, code of ethics, governance, and decision making.
2. Administrative information, such as training during normal hours or use of overtime and budgeting for the simulation training program.
3. Course directors and facilitators.
4. Evaluation methods, feedback methods, and remediation.
5. Equipment and supplies.

The SSH document goes into much greater detail, but these are a good start. If a more exhaustive approach is desired, then the full document should be consulted.

This is a simple guide for a Simulation Training Design Guide and not a comprehensive list because each center has different treatment equipment and software, and procedures. It is incumbent upon each facility to critically evaluate its procedures and design Simulation Training to maximize the integrity of patient care. A sample development process is shown below.

## 7 SAMPLE SIMULATION TRAINING SCENARIO DEVELOPMENT

1. Clinical or Technical Process
  - Clinical process, such as 4DCT simulation
  - Technical processes such as HDR source exchange
2. Type of simulation
  - Documents or simulated records in the Electronic Medical Record system with known errors.
  - Simulated process with phantom(s)
  - Virtual simulation environment
3. Staff involved
4. Frequency of training
5. Course developers
6. Equipment and supplies needed
7. Evaluation method
8. Feedback method
9. Remediation of failing performance

The patient process, as shown below, can be used as a guide for identifying scenarios.

1. Patient diagnosis by attending physicians.
2. Do the Radiologist and Radiation Oncologist agree?
3. Are the diagnostic scans correct and sufficient?
4. Are the scans covering the correct anatomy?
5. Do the treatment planning scans (a) cover the anatomy, (b) correct scans, c) have the correct slice thickness and separation?
6. Has the scan set been imported to the correct planning system? Is the treatment planner credentialed and trained?
7. Is the final plan appropriate: (a) fulfill the prescription, (b) are the beam geometry and energies appropriate, (c) is the tumor dose delivery correct and are the normal tissues spared? (d) has the plan been checked by the planner and second planner, and by a Physicist? Has the plan been approved by the attending radiation oncologist?
8. Has the plan been exported to the correct treatment unit? Has the treatment unit's performance been verified? Have the radiation therapists checked the plan on the treatment unit?
9. Has the correct patient been brought to the treatment unit? Is the entire treatment team present? Has the patient's treatment position and setup been correctly verified? For multiple lesions, are the lesions treated in the correct sequence with the correct isocenter/beam geometry?
10. Has the patient received the correct number of fractions?

As mentioned earlier, data from incident learning systems such as ROILS can also be a good source to identify topics for simulation training.

One area of frequent errors is the treatment plan. There are various QC processes that can be utilized to catch the errors in a treatment plan, as described in AAPM TG 275^39^ and AAPM MPPG 11.a.^40^ A test patient can be created in the electronic medical record system and the treatment planning system by anonymizing an existing patient. A set of documents that contains known errors is put into the Electronic Medical Record (EMR). For example, the prescription in the plan does not match the prescription in the EMR, oral contrast density is not overridden, incorrect bolus thickness, structures that are missing contours on some slices, incorrect documentation of isocenter shift, and incorrect laterality on consent. AAPM Working Group on the Prevention of Errors has produced a series of test patients with known errors that can be used for this kind of simulation training^41^

Another example of a simulation scenario is performing a seed implant on a prostate phantom. Phantoms are commercially available (CIRS, for example) or can be manufactured in‐house.^42^

A simulation training program could be developed in a staged manner by gradually adding more complex materials. The use of paper‐based exercises could be the first step, with scenarios taken from a department incident learning system or a national database such as ROILS. Hands‐on scenarios could then be developed. These could range from simple situations, such as simulating a stuck HDR brachytherapy source while the dummy source is connected during a source exchange, to more complex procedures, such as end‐to‐end simulations of ion chamber calibration or commissioning of a Linac. A final step could be an investment in a virtual reality system wherein the user can be given the opportunity to (safely) learn from the experience of having made mistakes and understanding the consequences.

## 8 CONCLUSIONS

There are several practical options for introducing simulation training into the radiation oncology clinic, including paper‐based exercises, instructional videos, and virtual reality, all of which can be associated with participant evaluation. By adopting some of the approaches implemented in high reliability industries, a well‐thought‐out simulation training program will address the weaknesses of our current approach to developing and maintaining competency. This is particularly evident in the lack of simulation training in residency programs and the omission of simulation training in the recommended essentials and guidelines for clinical medical physics residency training programs (AAPM Report 249).^43^ The key benefits of moving towards simulation training include:

1. Training in a particular technique or process can be repeated as often as necessary.
2. Training takes place in the absence of clinical pressures.
3. Standardization of training material reduces confusion for participants and is acknowledged to enhance safety.

System failures can be realistically demonstrated in strong contrast to “in the field” training, which is our current approach. AAPM continues to explore, develop, and promote tools for simulation training for the benefit of the radiotherapy community and the patients it serves.

## AUTHOR CONTRIBUTIONS

All authors contributed to early drafts of this report. Final editing and revisions were done by Michael Schell, Andrew Beavis, and Daniel Pavord.

## CONFLICT OF INTEREST STATEMENT

The corresponding author has reviewed the required Conflict of Interest statement on file for each author and determined that disclosure of potential Conflicts of Interest is an adequate management plan. The authors listed below attest that they have no potential Conflicts of Interest related to the subject matter or materials presented in this document. The author listed below disclose the following potential Conflict(s) of Interest related to the subject matter or materials presented in this document.

Andrew Beavis is the Chief Scientific Officer for Vertual.

## References

[1] High‐reliability health care: getting there from here. Millbank Q. 2013;91(3):459‐490. doi: 10.1111/1468‐0009.12023 10.1111/1468-0009.12023 PMC3790522 24028696

[2] Institute of Medicine (US) Committee on Quality of Health Care in America , Kohn LT , Corrigan JM , Donaldson MS , eds. To Err is Human: Building a Safer Health System. Washington (DC): National Academies Press (US); 2000. 25077248

[3] https://www.cihi.ca/en/patient‐harm‐in‐canadian‐hospitals‐it‐does‐happen, accessed 1‐13‐2024

[4] Radiation Therapy Risk Profile WHO/IER/PSP/2008.12 © World Health Organization, 2008.

[5] James JT . A new, evidence‐based estimate of patient harms associated with hospital care. J Patient Saf. 2013;9:122‐128. doi: 10.1097/PTS.0b013e3182948a69 23860193 10.1097/PTS.0b013e3182948a69

[6] Weick KE . Organizational culture as a source of high reliability. Calif Manage Rev. 1987;29:112‐127. doi: 10.2307/41165243

[7] Friedrich A , Managing complex systems in perioperative medicine. Int Anesthesiol Clin. 2009;47(4):xi. doi: 10.1097/AIA.0b013e3181b47b24 19820475 10.1097/AIA.0b013e3181b47b24

[8] Abdulla A , Schell KR , Schell MC . Comparing the evolution of risk culture in radiation oncology, aviation, and nuclear power. J Patient Saf. 2020;16(4):e352‐e358. doi: 10.1097/PTS.0000000000000560 30608909 PMC7678666

[9] Aviation Statistics [database online]. Washington. DC: National Transportation Safety Board; 2018.

[10] Sovacool BK . The costs of failure: a preliminary assessment of major energy accidents, 1907–2007. Energy Policy. 2008;36:1802‐1820. doi: 10.1016/j.enpol.2008.01.040

[11] James JT . A new, evidence‐based estimate of patient harms associated with hospital care. J Patient Saf. 2013;9:122‐128. doi: 10.1097/PTS.0b013e3182948a69 23860193 10.1097/PTS.0b013e3182948a69

[12] Huq SM , Fraass BA , Dunscombe PB , et al. The report of task group 100 of the AAPM: application of risk analysis methods to radiation therapy quality management. Med Phys. 2016;43:4209‐4262. doi: 10.1118/1.4947547 27370140 10.1118/1.4947547 PMC4985013

[13] Ezzell G , Chera B , Dicker A , et al. Common error pathways seen in the RO‐ILS data that demonstrate opportunities for improving treatment safety. Pract Radiat Oncol. 2018;8(2):123‐132. doi: 10.1016/j.prro.2017.10.007 29329998 10.1016/j.prro.2017.10.007

[14] Lane JL , Slavin S , Ziv A . Simulation in medical education: a review. Simul Gaming. 2001;32:297. doi: 10.1177/104687810103200302

[15] Email Rodney , Martz Sr , Technical Specialist, Pilot Information Center. AOPA. August 6, 2015.

[16] Helmreich RL , Merritt AC , Wilhelm JA . The evolution of crew resource management training in commercial aviation (PDF). Int J Aviat Psychol. 1999;9(1):19‐32. doi: 10.1207/s15327108ijap0901_2 11541445 10.1207/s15327108ijap0901_2

[17] https://www.nytimes.com/2019/06/01/business/boeing‐737‐max‐crash.html

[18] Travis G . How the Boeing 737 Max Disaster Looks to a Software Developer. IEEE Spectrum. 2019;18.

[19] Kemeny JG . Report of The President's Commission on the Accident at Three Mile Island: The Need for Change: The Legacy of TMI. The Commission; 1979. ISBN 0‐935758‐00‐3.

[20] Cass Stephen . Houston, we have a solution, part 2. IEEE. April 1, 2005 Retrieved August 31, 2019.

[21] Lovell Jim , Kluger Jeffrey , (2000) [1994].Lost Moon: The Perilous Voyage of Apollo 13. Boston: Houghton Mifflin. ISBN 978‐0‐618‐05665‐1.

[22] Okuda Y , Quinones J . The use of simulation in the education of emergency care providers for cardiac emergencies. Int J Emerg Med. 2008;1(2):73‐77. doi: 10.1007/s12245‐008‐0034‐2 19384655 10.1007/s12245-008-0034-2 PMC2657247

[23] Satava RM . The revolution in medical education‐the role of simulation. J Grad Med Educ. 2009;1(2):172‐175. doi: 10.4300/JGME-D-09-00075.1 21975972 PMC2931262

[24] Runciman WB , Merry AF . The wondrous story of anesthesia. In: A Brief History of the Patient Safety Movement in Anaesthesia, 2013:541‐556.

[25] Cooper JB , Taqueti VR . A brief history of the development of mannequin simulators for clinical education and training. Qual Saf Health Care. 2005;14(1):72. doi: 10.1136/qshc.2004.009886 PMC1765785 15465949

[26] Rosen MA , Salas E , Silvestri S , Wu TS , Lazzara EH . A measurement tool for simulation‐based training in emergency medicine: the simulation module for assessment of resident targeted event responses (SMARTER) approach. Simul Healthc. 2008;3(3):170‐179. doi:10.1097/SIH.0b013e318173038d. Fall. 19088661 10.1097/SIH.0b013e318173038d

[27] Bracq MS , Michinov E , Jannin P . Virtual reality simulation in nontechnical skills training for healthcare professionals: a systematic review. Simul Healthc. 2019;14(3):188‐194. doi: 10.1097/SIH.0000000000000347 30601464

[28] Brazil V , Purdy EI , Bajaj K . Connecting simulation and quality improvement: how can healthcare simulation really improve patient care?. BMJ Qual Saf. 2019;28:862‐865. doi:10.1136/bmjqs-2019-009767 31320498

[29] Beavis AW . The opportunities of computer simulation training in radiation therapy. J Med Radiat Sci. 2018;65(2):77‐79. doi: 10.1002/jmrs.283 29864247 10.1002/jmrs.283 PMC5986043

[30] Phillips R , Ward JW , Page L , et al. Virtual reality training for radiotherapy becomes a reality. Stud Health Technol Inform. 2008;132:366‐371. 18391323

[31] VERT final project report., http://www.sor.org/learning/document‐library/vert‐final‐project‐report 2010.

[32] Beavis A , Ward J . WE‐G‐BRA‐04: the development of a virtual reality dosimetry training platform for physics training. Med Phys. 2012;39(6Part28):3969. doi: 10.1118/1.4736199 10.1118/1.4736199 28519646

[33] Beavis AW , Brisband C , Clark CH , et al, (2024, July). Cloud based computer simulation training for guided, remote radiation therapy training and assessment. In AAPM 66th Annual Meeting & Exhibition. AAPM.

[34] Jimenez YA , Cumming S , Wang W , et al. Patient education using virtual reality increases knowledge and positive experience for breast cancer patients undergoing radiation therapy. Support Care Cancer. 2018;26:2879‐2888. doi:10.1007/s00520-018-4114-4 29536200

[35] Buliński K , Kuszewski T , Wnuk K , Braziewicz J , Ślosarek K . Uncertainties in the measurement of relative doses in radiotherapy. J Pol Soc Med Phys. 2021;27(1):1‐9. 10.2478/pjmpe-2021-0001

[36] Pavord DC , Birnbaum S , Boccuzzi D , et al. A report from the AAPM subcommittee on guidelines for competency evaluation for clinical medical physicists in radiation oncology. J Appl Clin Med Phys. 2016;17:3‐14. doi:10.1120/jacmp.v17i4.5804 PMC5690041 27455473

[37] Giuliani M , Gillan C , Wong O , et al. Evaluation of high‐fidelity simulation training in radiation oncology using an outcomes logic model. Radiat Oncol. 2014;9:189. doi: 10.1186/1748-717X-9-189 25169674 PMC4155118

[38] https://ssih.org/sites/default/files/2025‐04/Policy%20Manual%20Template%20v2021.pdf, accessed 1‐13‐2024

[39] Ford E , Conroy L , Dong L , et al. Strategies for effective physics plan and chart review in radiation therapy: report of AAPM Task Group 275. Med Phys. 2020;47(6):e236‐e272. doi: 10.1002/mp.14030 31967655 10.1002/mp.14030 PMC9012523

[40] Xia P , Sintay BJ , Colussi VC , et al. Medical Physics Practice Guideline (MPPG) 11. a: plan and chart review in external beam radiotherapy and brachytherapy. J Appl Clin Med Phys. 2021;22(9):4‐19. doi: 10.1002/acm2.13366 10.1002/acm2.13366 PMC8425907 34342124

[41] Accessed 11‐17‐2024https://www.aapm.org/QualitySafety/ErrorTraining.asp

[42] Chiu T , Xiong Z , Parsons D , Folkert MR , Medin PM , Hrycushko B . Low‐cost 3D print–based phantom fabrication to facilitate interstitial prostate brachytherapy training program. Brachytherapy. 2020;19(6):800‐811. doi: 10.1016/j.brachy.2020.06.015 32690386 10.1016/j.brachy.2020.06.015

[43] Prisciandaro JI , Willis CE , Burmeister JW , et al. Essentials and guidelines for clinical medical physics residency training programs: executive summary of AAPM Report Number 249. J Appl Clin Med Phys. 2014;15(3):4‐13. doi: 10.1120/jacmp.v15i3.4763 10.1120/jacmp.v15i3.4763 PMC5711071 24892354

